# Role of integrin expression in the prediction of response to vedolizumab: A prospective real‐life multicentre cohort study

**DOI:** 10.1002/ctm2.769

**Published:** 2022-04-05

**Authors:** Cara De Galan, Gerard Bryan Gonzales, Sophie Van Welden, Simon Jan Tavernier, Triana Lobaton, Wouter Van Moerkercke, Beatrijs Strubbe, Harald Peeters, Elisabeth Macken, Martine De Vos, Debby Laukens, Pieter Hindryckx

**Affiliations:** ^1^ Department of Internal Medicine and Paediatrics Ghent University Ghent Belgium; ^2^ Ghent Gut Inflammation Group (GGIG) Ghent University Ghent Belgium; ^3^ VIB Center for Inflammation Research Ghent Belgium; ^4^ Primary Immune Deficiency Research Lab Centre for Primary Immunodeficiency Ghent Jeffrey Model Diagnosis and Research Centre Ghent University Hospital Ghent Belgium; ^5^ Department of Gastroenterology Ghent University Hospital Ghent Belgium; ^6^ Department of Gastroenterology AZ Groeninge Kortrijk Belgium; ^7^ Department of Gastroenterology AZ Sint‐Lucas Ghent Ghent Belgium; ^8^ Department of Gastroenterology University Hospital Antwerp Antwerp Belgium

Dear editor,

Despite the growing number of treatments approved for inflammatory bowel disease (IBD), patient outcomes can still be unsatisfactory due to highly variable response rates.^1^ Vedolizumab, first alternative biological for anti‐tumour necrosis factor (TNF) in IBD management, targets α4β7 integrin heterodimers on circulatory T cells and inhibits their binding to mucosal addressin cell adhesion molecule 1 (MAdCAM‐1). Despite its excellent benefit‐risk profile, only 40–60% of IBD patients will respond, emphasizing the need for personalized medicine.^1^, ^2^ We performed the largest real‐life prospective multicentre cohort study reported to date (*n* = 71) with serial sample collection at week (w) 0, 2, 6, 10 (only for CD) and 14 (Figure 1), to evaluate whether integrin expression profiles on circulatory T cells are potential biomarkers of vedolizumab response. The definition of response is described in Table S1.

**FIGURE 1:**
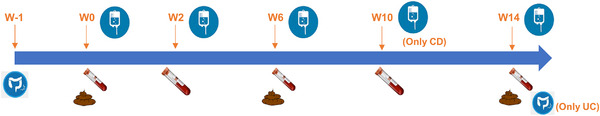
Schematic overview of sample collection during the clinical study. Endoscopy was performed before vedolizumab initiation to validate active disease. At baseline, stool and blood samples were collected which was repeated at weeks (w) 2, 6, 10 (only for CD patients) and 14. At w14, the clinical and biochemical response was assessed. In UC patients, an additional endoscopy was performed at w14 according to the national reimbursement criteria (which entails previous failure of immunosuppressants)

Although vedolizumab only targets α4β7, other dimers contribute to lymphocyte infiltration in the gut mucosa of IBD patients, including α4β1 and αEβ7.^2^, ^3^ Therefore, a highly qualitative flow cytometry analysis of α4, αE, β1 and β7 (Supplementary methods and Figures [Link], [Link], [Link], [Link]) was performed on peripheral blood mononuclear cells of 44 ulcerative colitis (UC) and 27 Crohn's disease (CD) patients with moderate‐to‐severe disease, who initiated vedolizumab as part of their conventional treatment plan (Table S2). Response rates at w14 were similar as previously reported (Table S3)^1^, ^2^ and were not linked with age, gender, age at diagnosis, baseline C‐reactive protein (CRP) and previous anti‐TNF use. The biochemical response rate was significantly higher in UC patients with left‐sided colitis than in those with pancolitis (66.7% vs. 33.3%; *p* = .015), and similar trend could be observed in endoscopic responders (72.0% vs. 24.0%; *p* = .066), partially confirming the data of Scarozza et al.^4^ Current or previous smoking was associated with clinical and endoscopic response in UC (*p* = .040 and *p* = .039, respectively) (Table 1).

**TABLE 1:** Characteristics of responder and non‐responder UC and CD patients

		Clinical			Biochemical			Endoscopic		
		R	NR	*p*	R	NR	*p*	R	NR	*p*
**UC**	**Age** (years), mean [min–max]	43 [20–76]	39 [18–73]	ns	46 [21–76]	32 [18–55]	ns	45 [20–76]	39 [18–73]	ns
	**Gender**, *n* [%]			ns			ns			ns
	Female	17 [56.7]	8 [57.1]		13 [61.9]	5 [62.5]		14 [56.0]	9 [56.3]	
	Male	13 [43.3]	6 [42.9]		8 [38.1]	3 [37.5]		11 [44.0]	7 [43.8]	
	**Age at diagnosis**, mean [min–max]	33 [11–68]	32 [14–67]	ns	38 [19–68]	20 [14–32]	ns	35 [16–68]	31 [14–67]	ns
	**Smoking** ^†^, *n* [%]			*			ns			*
	Yes	9 [30.0]	0 [0.0]		3 [14.3]	0 [0.0]		7 [28.0]	1 [6.3]	
	No	21 [70.0]	14 [100.0]		18 [85.7]	8 [100.0]		18 [72.0]	15 [93.8]	
	**Baseline CRP**, median [IQR]	25 [4–64]	15 [8–29]	ns	11 [4–61]	24 [21–35]	ns	34 [7–64]	13 [2–24]	ns
	**Extent**, *n* [%]			ns			*			.066
	E1	1 [3.3]	1 [7.1]		0 [0.0]	1 [12.5]		1 [4.0]	1 [6.2]	
	E2	20 [66.7]	6 [42.9]		14 [66.7]	1 [12.5]		18 [72.0]	7 [43.8]	
	E3	9 [30.0]	7 [50.0]		7 [33.3]	6 [75.0]		6 [24.0]	8 [50.0]	
	**Previous anti‐TNF use** [%]	17 [56.7]	9 [64.3]	ns	11 [52.4]	7 [87.5]	ns	14 [56.0]	10 [62.5]	ns
**CD**	**Age** (years), mean [min–max]	44 [24–65]	37 [20–68]	ns	43 [24–65]	40 [27–68]	ns			
	**Gender**, *n* [%]			ns			ns			
	Female	7 [50.0]	8 [61.5]		8 [53.3]	7 [58.3]				
	Male	7 [50.0]	5 [38.5]		7 [46.7]	5 [41.7]				
	**Age at diagnosis**, mean [min–max]	36 [11–64]	28 [15–57]	ns	35 [16–64]	30 [15–57]	ns			
	**Smoking** ^†^, *n* [%]			ns			ns			
	Yes	4 [28.6]	5 [38.5]		4 [26.7]	5 [41.7]				
	No	10 [71.4]	8 [61.5]		11 [73.3]	7 [58.3]				
	**Baseline CRP**, median [IQR]	57 [12–228]	15 [3–17]	ns	34 [7–119]	10 [3–51]	ns			
	**Extent**, *n* [%]			ns			ns			
	L1	7 [50.0]	5 [38.5]		7 [46.7]	5 [41.7]				
	L2	3 [21.4]	7 [53.8]		3 [20.0]	7 [58.3]				
	L3	4 [28.6]	1 [7.7]		5 [33.3]	0 [0.0]				
	L4	0 [0.0]	0 [0.0]		0 [0.0]	0 [0.0]				
	**Previous anti‐TNF use** [%]	6 [42.9]	6 [46.2]	ns	6 [40.0]	5 [41.7]	ns			

^†^ Current and previous history of smoking.

CD: Crohn's disease, CRP: C‐reactive protein; IQR: interquartile range; R: responders; NR: non‐responders; ns: not significant; UC: ulcerative colitis.

* *p* < .05.

The number of pre‐treatment circulatory CD4^+^α4β7^+^ T cells were higher in UC patients with clinical and biochemical response (*p* = .031 and *p* = .004, respectively) (Figure 2A–D) and CD. In CD however, baseline circulatory CD4^+^α4β1^+^ T cell numbers were higher in biochemical non‐responders (*p* = .009) (Figure 2E–G), while the number of baseline CD8^+^α4β7^+^, CD8^+^α4β1^+^, CD4^+^ and CD8^+^αEβ7^+^ T cells were similar between responders and non‐responders. These results suggest that the α4β1‐vascular cell adhesion molecule 1 gut‐homing pathway might drive vedolizumab non‐response in CD.

**FIGURE 2:**
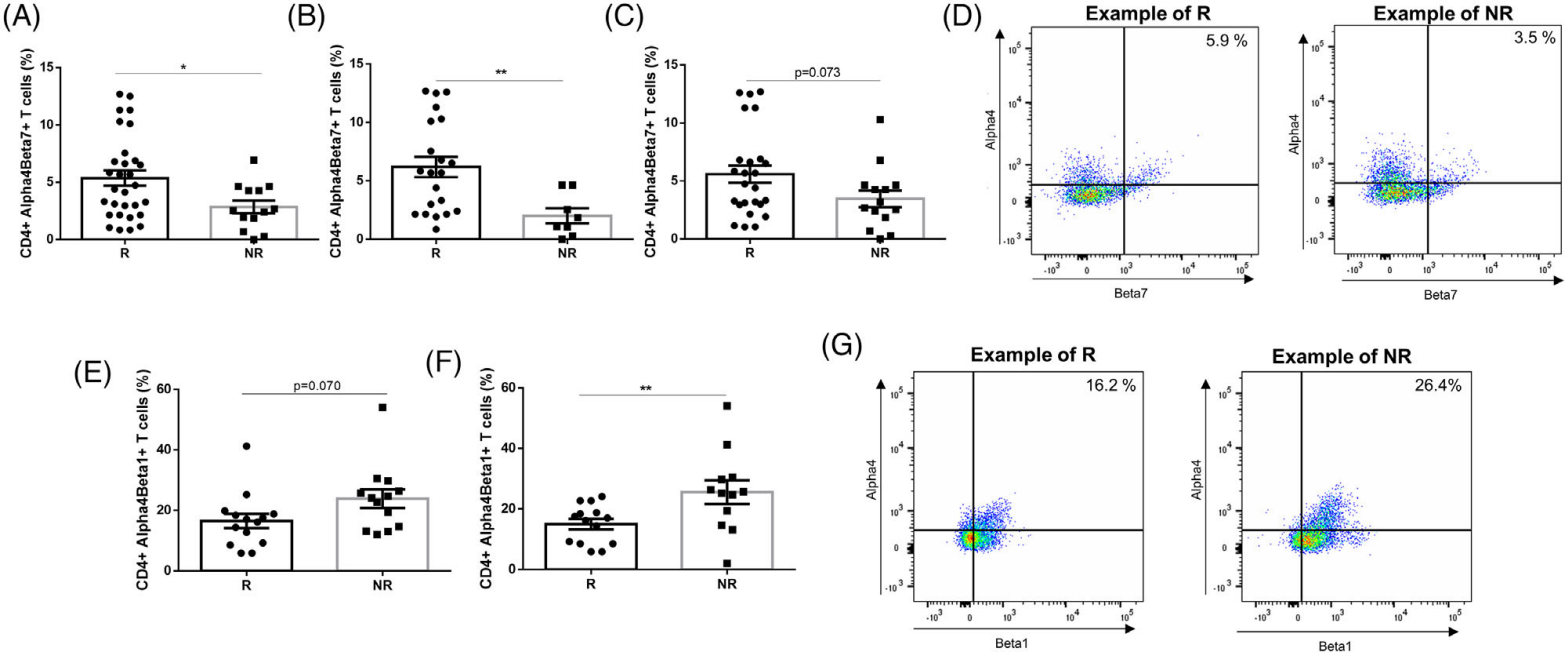
High abundance of baseline CD4^+^α4β7^+^ T cells in UC and low abundance of CD4^+^α4β1^+^ T cells in CD are positively associated with response to vedolizumab. The number of CD4^+^α4β7^+^ T cells significantly differs between responder (R) and non‐responder (NR) UC patients on (A) clinical, (B) biochemical and (C) endoscopic level. (D) Representative dot plots of α4 and β7 expression in randomly selected responder and non‐responder UC patients. The number of CD4^+^α4β1^+^ T cells also differs between responder and non‐responder CD patients on (E) clinical and (F) biochemical level. (G) Representative dot plots of α4 and β1 expression in randomly selected responder and non‐responder CD patients. Bar charts indicate the mean and standard error of the mean (SEM). *p < .05, **p < .01

Delta change differences between w0 and w2, w0 and w6, w2 and w6 (Figures [Link], [Link], [Link]) and mean fluorescence intensity of all investigated integrins at baseline (Figures [Link], [Link], [Link], [Link], [Link], [Link]) did not differ between responders and non‐responders in the entire IBD nor in the UC and CD cohort.

Since the abundance of αE^+^ T cells is higher in the ileum compared to the colon,^5^ we focused on integrin expression in patients stratified by disease location. Using this strategy, the number of baseline CD4^+^ and CD8^+^αEβ7^+^ T cells were positively associated with clinical response in CD patients with ileal disease (*p* = .064 and *p* = .039, respectively) (Figure S14), indicating its possible pathogenic relevance for ileal disease. No significant differences could be identified related to other dimers (Figure S15).

Finally, data on integrin profiles were used to build a prognostic model together with previously identified clinical and biochemical markers of response to vedolizumab.^6^, ^7^, ^8^, ^9^, ^10^ In contrast to previous proof‐of‐concept studies, we were not able to confirm association with vedolizumab trough levels, soluble MAdCAM‐1, retinoic acid and albumin^6^, ^7^, ^8^, ^9^, ^10^ (Figures [Link], [Link], [Link], [Link]), possibly due to our w14 endpoint compared to previously reported w30 and w52 endpoints.^6^, ^7^, ^10^

In order to translate our observations to clinical application, we evaluated whether the number of CD4^+^α4β7^+^ and CD4^+^α4β1^+^ T cells at baseline are predictors of vedolizumab response. For UC, a predictive model could be created using elastic net regularized regression (EN) with a bootstrap validated area under the receiver operator curve (AUROC) of 91% [0.82–1.00]. In this model, baseline levels of CD4^+^α4β7^+^ T cells, CRP levels at baseline, smoking history, baseline levels of CD4^+^αEβ7^+^ T cells and CD4^+^α4β1^+^ T cells were the top 5 features selected. However, bootstrap validation indicated that these variables are not robust predictors, given their appearance in less than 60% of the 2000 bootstrap iterations (Figure 3A). Considering that 76% of UC patients responded to therapy in the training set, a predictive model for response will always return a high AUROC. A cross‐validation was performed to further validate these observations, in which an equal number of responders and non‐responders (*n* = 4) were placed into a training and validation set. As discussed above, when the model only gives 1 answer (responder), a high AUROC can still be achieved due to the response rate in our cohort; however, when we validate this AUROC with an equal number of responders and non‐responders, the AUROC is 75%, but random within the confidence interval and validation fails (Figure 3B).

**FIGURE 3:**
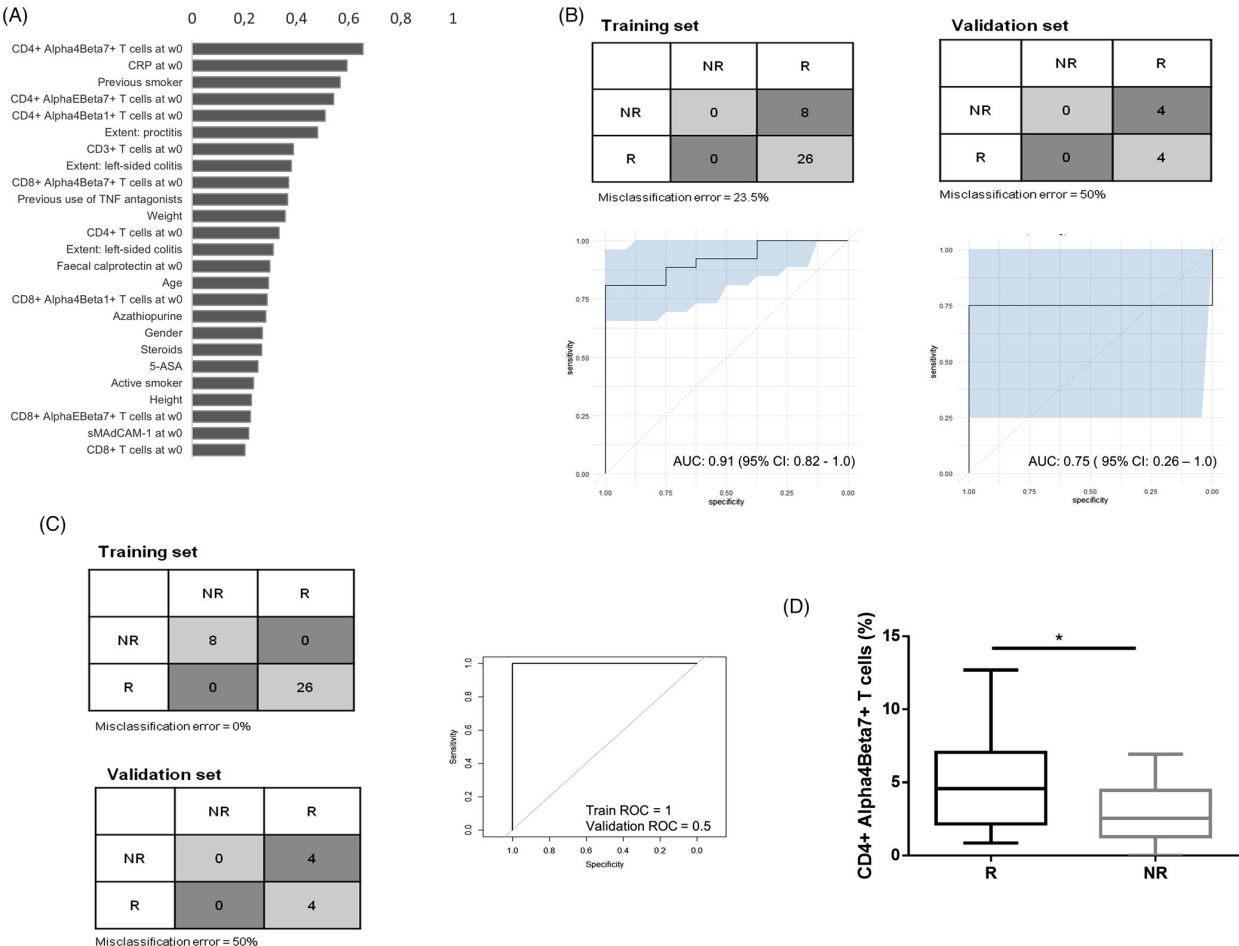
Predictive model development using elastic net regularized regression and random forest in the UC patient cohort. (A) Frequency of the features appeared in the 2000 generated models to differentiate between responders (R) and non‐responders (NR). (B) Confusion matrix of the training and validation set of the UC cohort with the complementary area under the receiver operator curve (AUROC) obtained with elastic net regularized regression (EN). (C) Confusion matrix of the training and validation set with the complementary AUROCs using random forest (RF). The rows in the confusion matrix indicate their response and the columns indicate their prediction. The complementary AUROC is find below the confusion matrix together with the confidence interval. (D) Boxplot of CD4^+^α4β7^+^ T cell abundance indicating the variation in the flow cytometry measurements with the interquartile range (IQR). *p < .05

To validate our EN model, random forest (RF) was employed. Similar to the EN model, flow cytometry data were unable to generate reliable predictions of response (AUROC = 50%, misclassification error = 50% in validation set) (Figure 3C). Although it can be inferred that the baseline number of CD4^+^α4β7^+^ T cells are statistically different in responders versus non‐responders, it cannot robustly predict vedolizumab response, which is probably due to large variation in the numbers of CD4^+^α4β7^+^ T cells, characteristic when quantifying low abundant cell types (Figure 3D).

In conclusion, we demonstrated that the high abundance of baseline CD4^+^α4β7^+^ T cells in UC and low abundance of baseline CD4^+^α4β1^+^ T cells in CD are positively associated with vedolizumab response. In addition, we provide further evidence that the high abundance of baseline CD4^+^ and CD8^+^ αEβ7^+^ T cells were positively associated with vedolizumab response in CD patients suffering from ileal disease, further confirming that response rates to vedolizumab may depend on disease location. Although the prognostic value of integrin phenotypes could not be validated, our study shows important blood immune cell heterogeneity in IBD and further supports the concept that the mechanism of action of vedolizumab is not exclusively related to inhibiting α4β7‐MAdCAM‐1 interaction.

## CONFLICT OF INTEREST

None of the authors have any conflicts of interest to declare.

## Supporting information

SUPPORTING INFORMATION

SUPPORTING INFORMATION

SUPPORTING INFORMATION

SUPPORTING INFORMATION

SUPPORTING INFORMATION

SUPPORTING INFORMATION

SUPPORTING INFORMATION

SUPPORTING INFORMATION

SUPPORTING INFORMATION

SUPPORTING INFORMATION

SUPPORTING INFORMATION

SUPPORTING INFORMATION

SUPPORTING INFORMATION

SUPPORTING INFORMATION

SUPPORTING INFORMATION

SUPPORTING INFORMATION

SUPPORTING INFORMATION

SUPPORTING INFORMATION

SUPPORTING INFORMATION

SUPPORTING INFORMATION

SUPPORTING INFORMATION

SUPPORTING INFORMATION

SUPPORTING INFORMATION
