# Supplementary figures and images for: Role of integrin expression in the prediction of response to vedolizumab: A prospective real‐life multicentre cohort study
Source: Clin Transl Med. 2022 Apr 5;12(4):e769. doi: 10.1002/ctm2.769 (PMC8982506; doi:10.1002/ctm2.769)

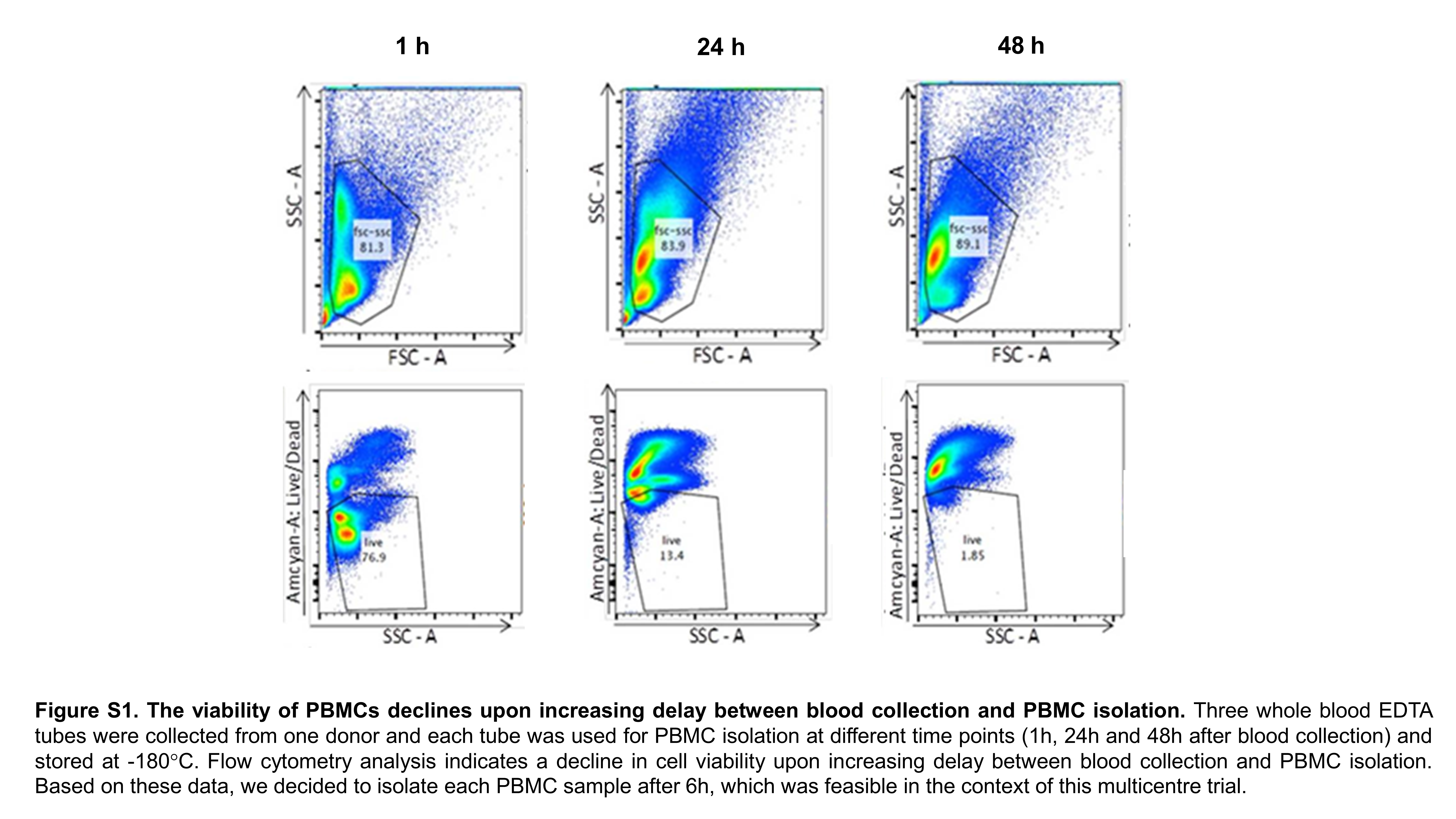

Supplement: Supplementary file 1 — SUPPORTING INFORMATION [file CTM2-12-e769-s015.tif]

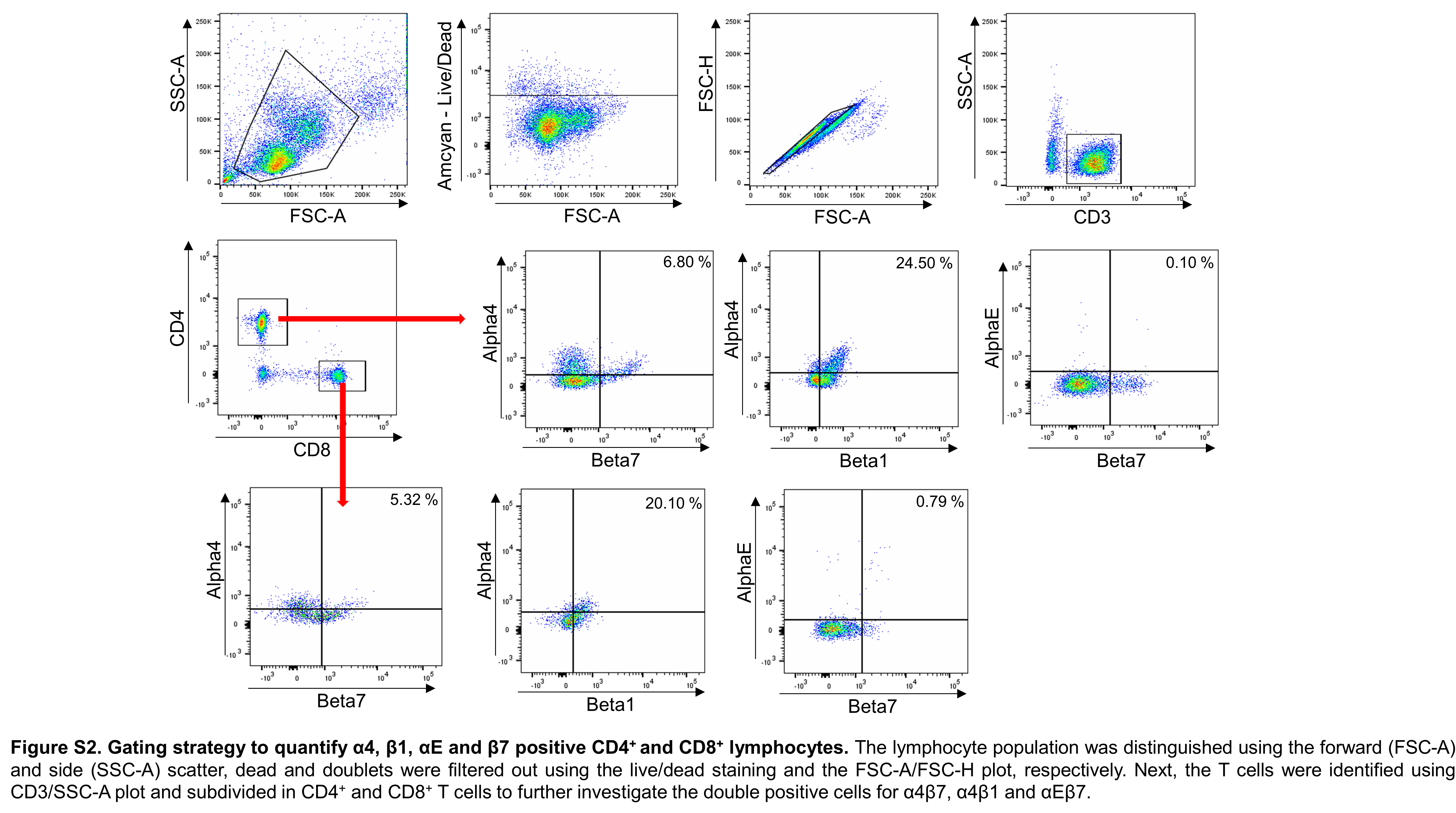

Supplement: Supplementary file 2 — SUPPORTING INFORMATION [file CTM2-12-e769-s023.tif]

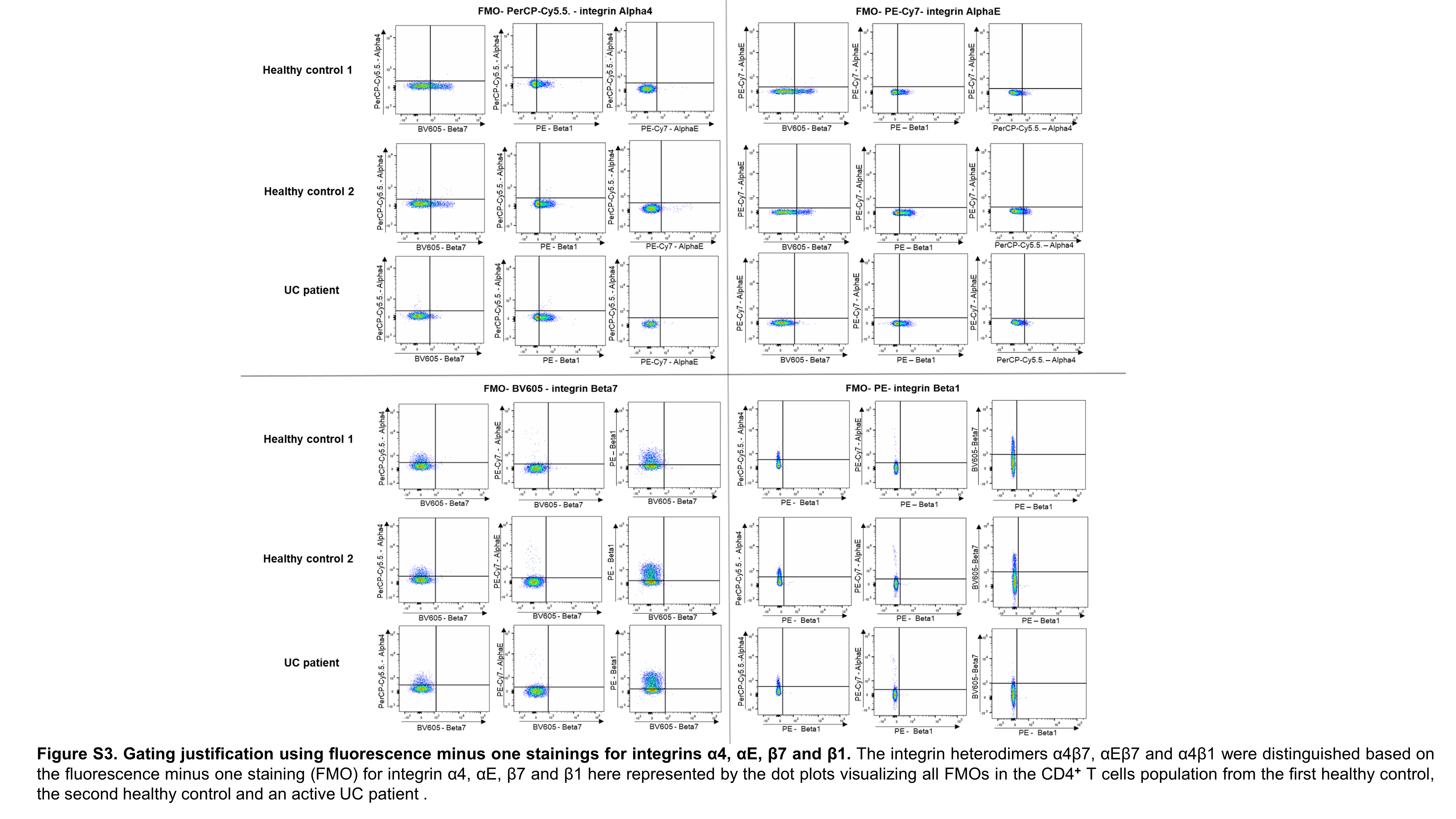

Supplement: Supplementary file 3 — SUPPORTING INFORMATION [file CTM2-12-e769-s002.tif]

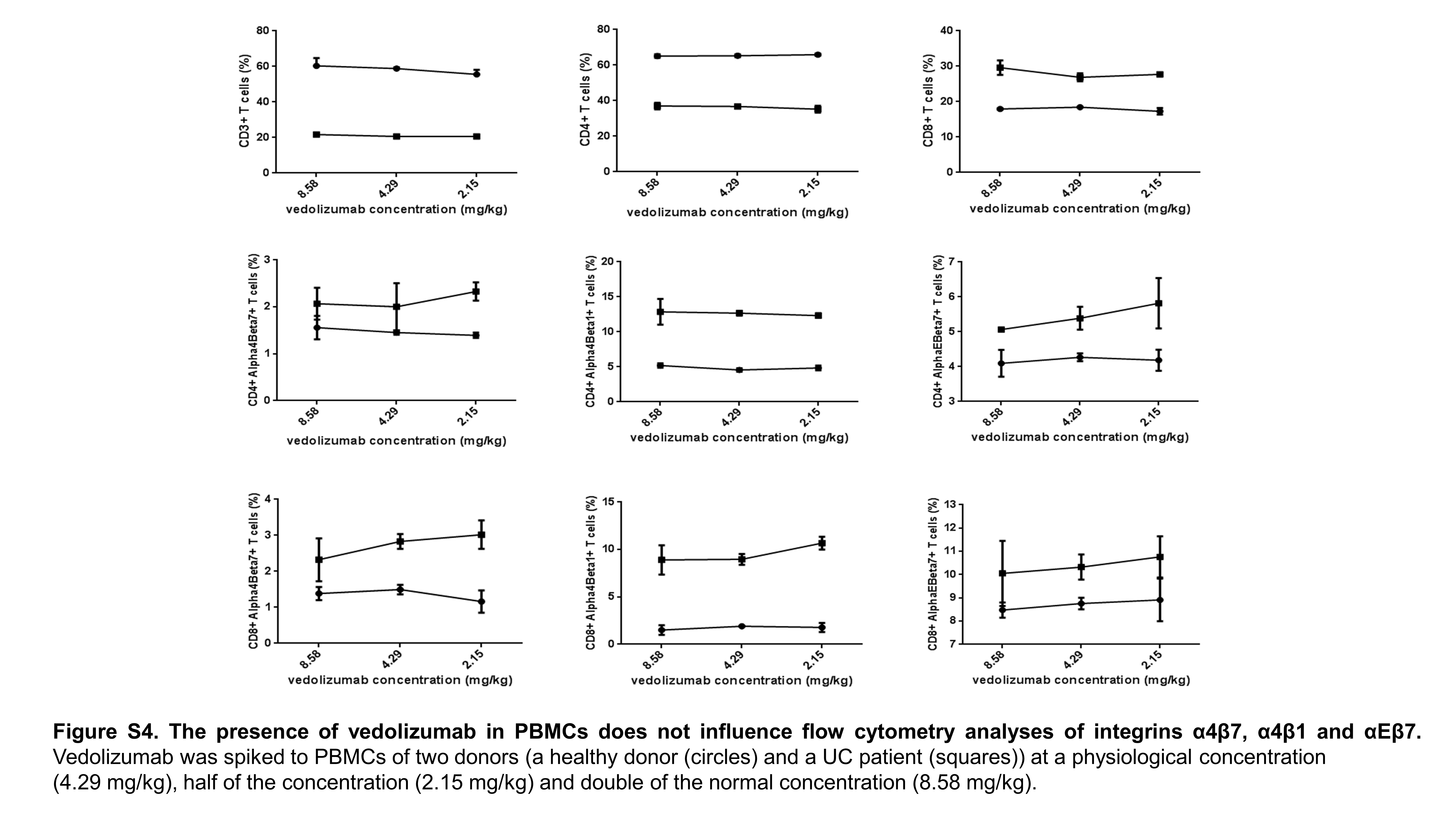

Supplement: Supplementary file 4 — SUPPORTING INFORMATION [file CTM2-12-e769-s012.tif]

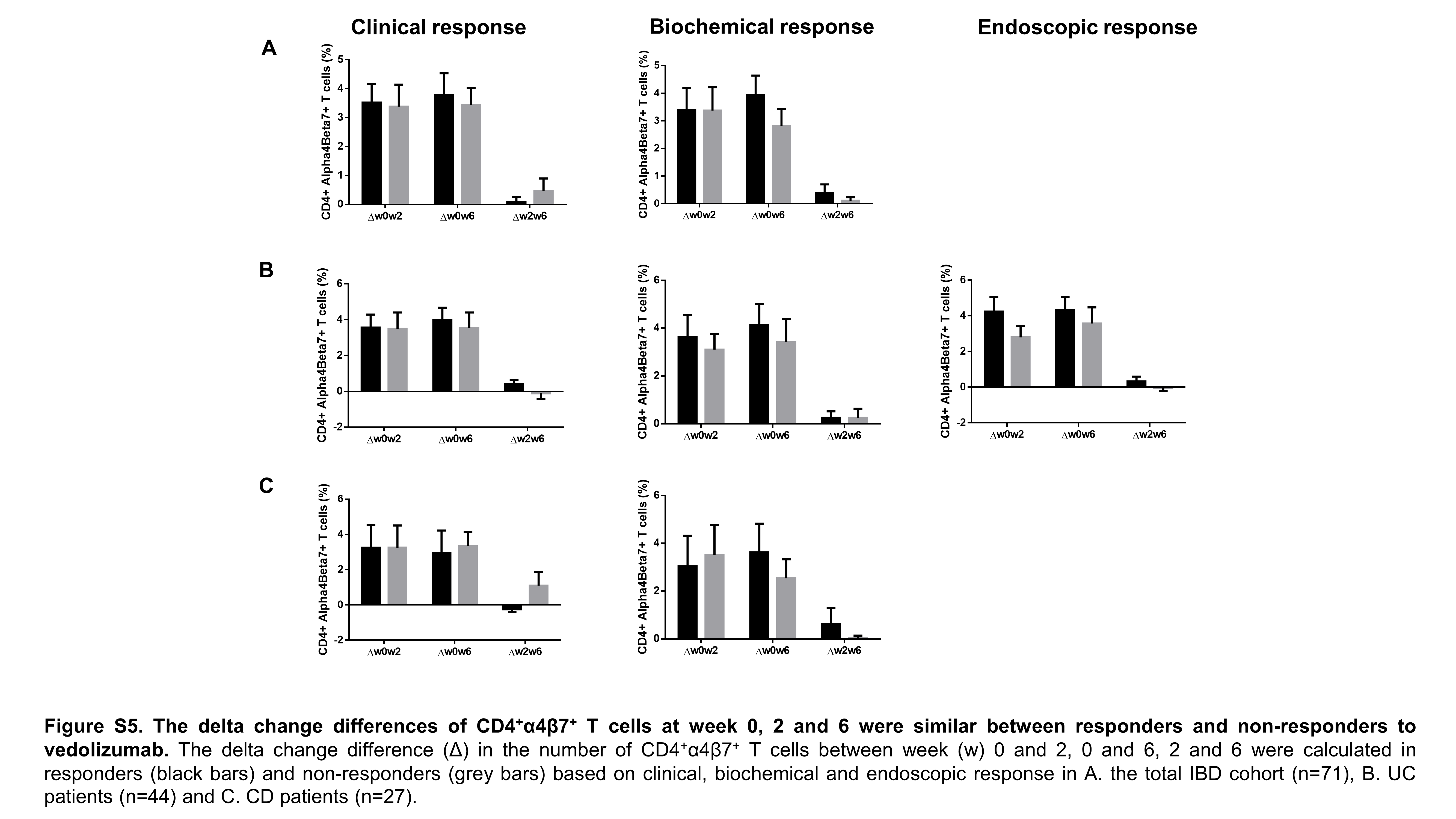

Supplement: Supplementary file 5 — SUPPORTING INFORMATION [file CTM2-12-e769-s007.tif]

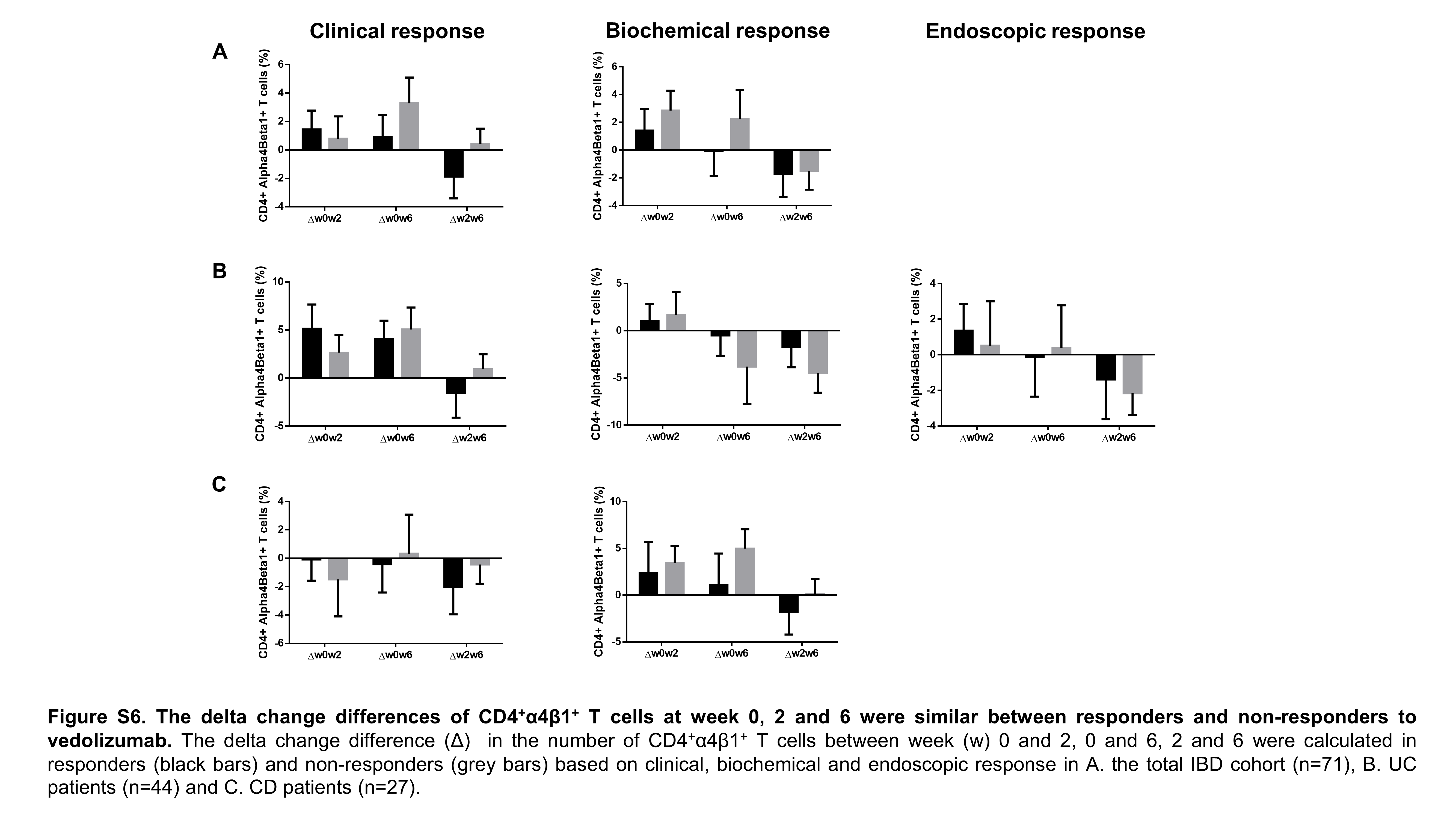

Supplement: Supplementary file 6 — SUPPORTING INFORMATION [file CTM2-12-e769-s006.tif]

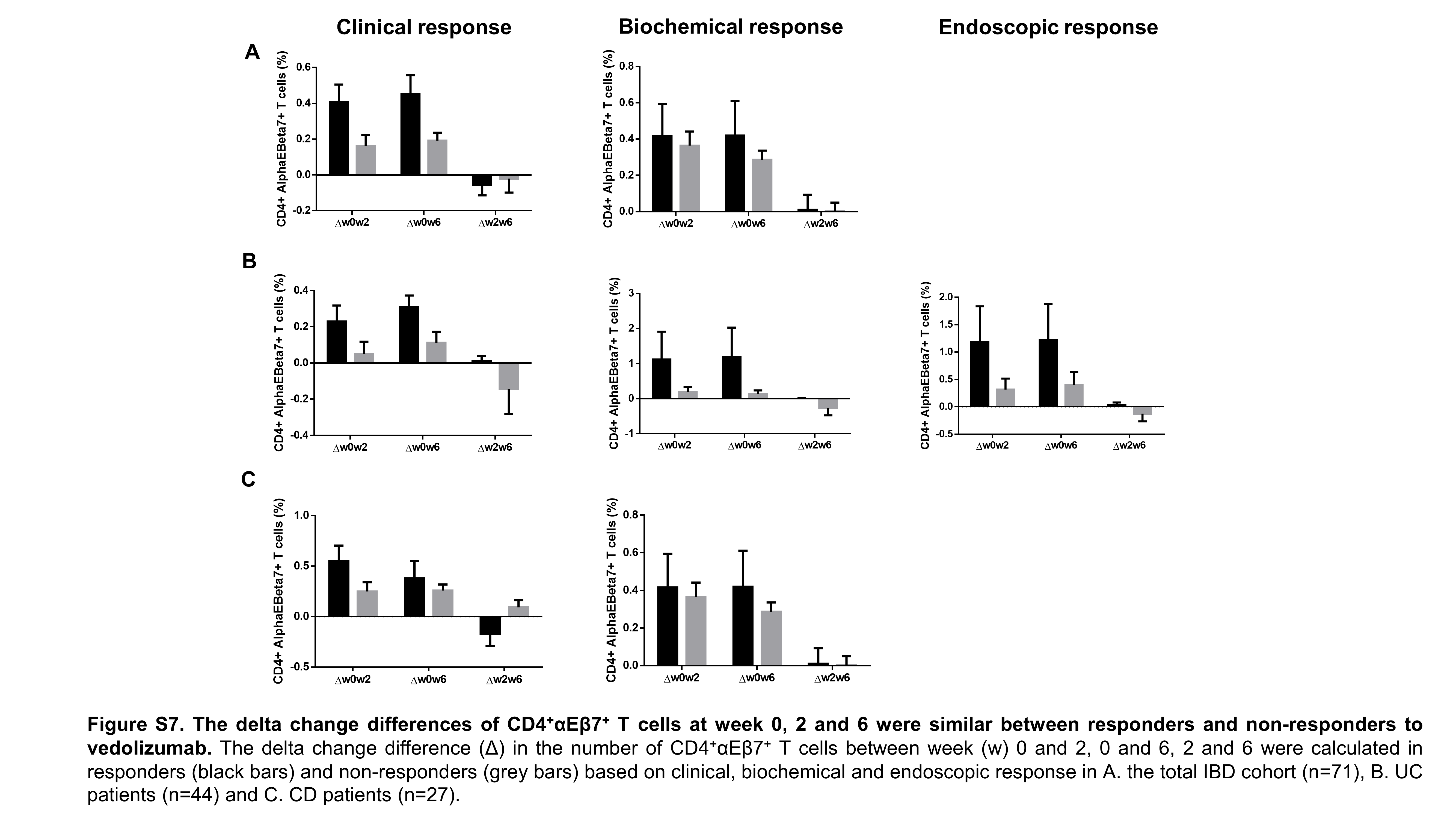

Supplement: Supplementary file 7 — SUPPORTING INFORMATION [file CTM2-12-e769-s014.tif]

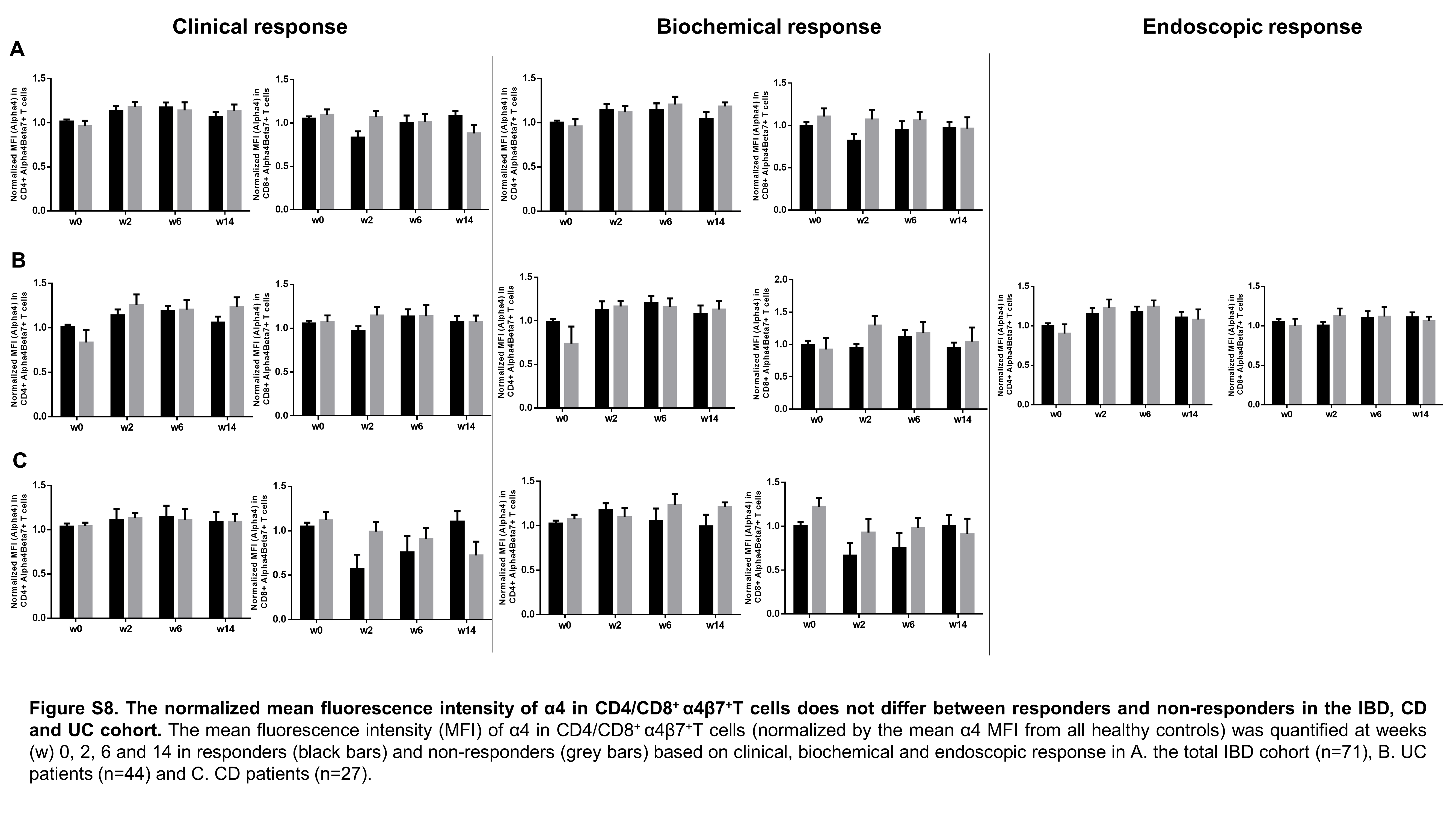

Supplement: Supplementary file 8 — SUPPORTING INFORMATION [file CTM2-12-e769-s001.tif]

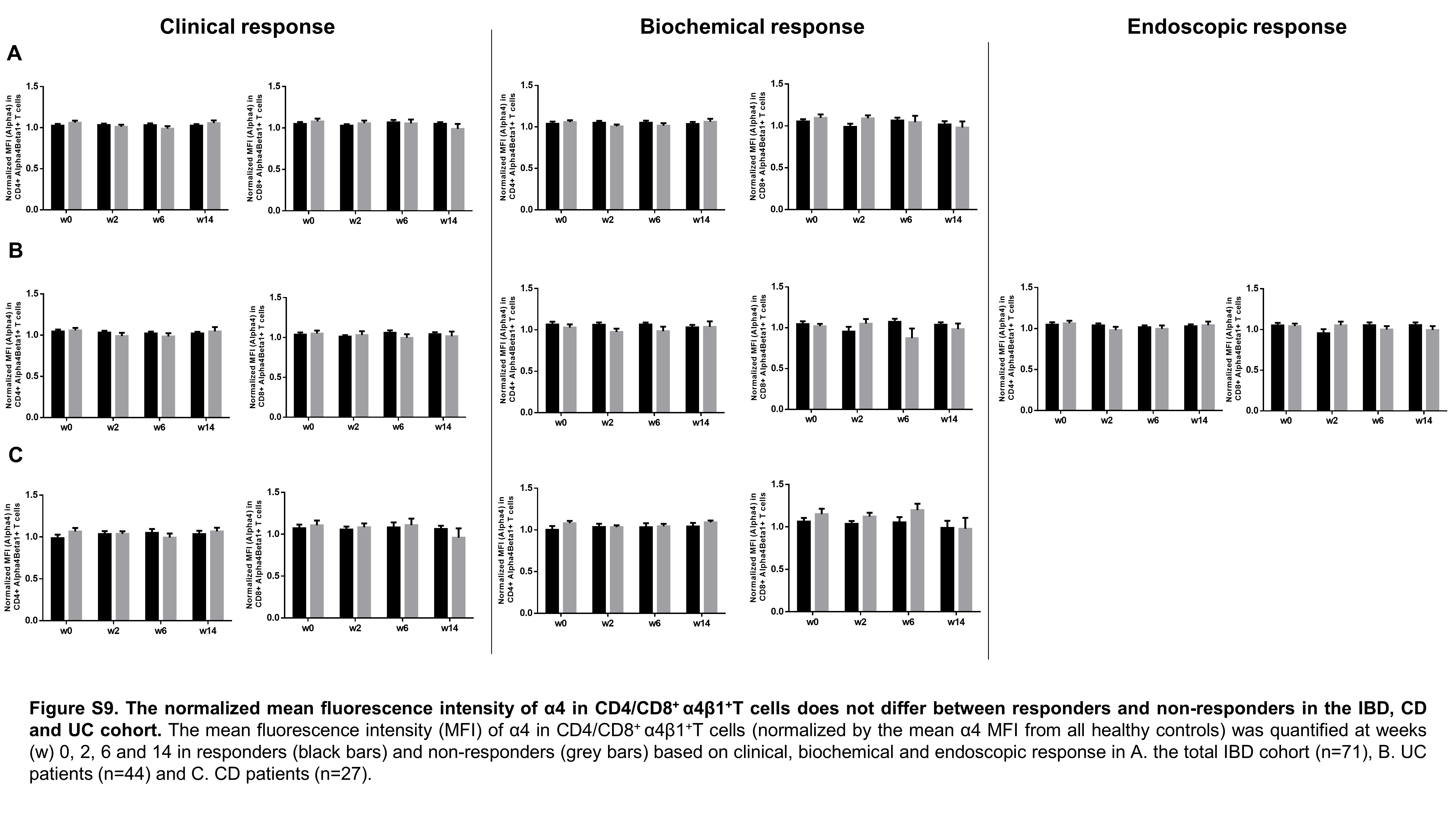

Supplement: Supplementary file 9 — SUPPORTING INFORMATION [file CTM2-12-e769-s017.tif]

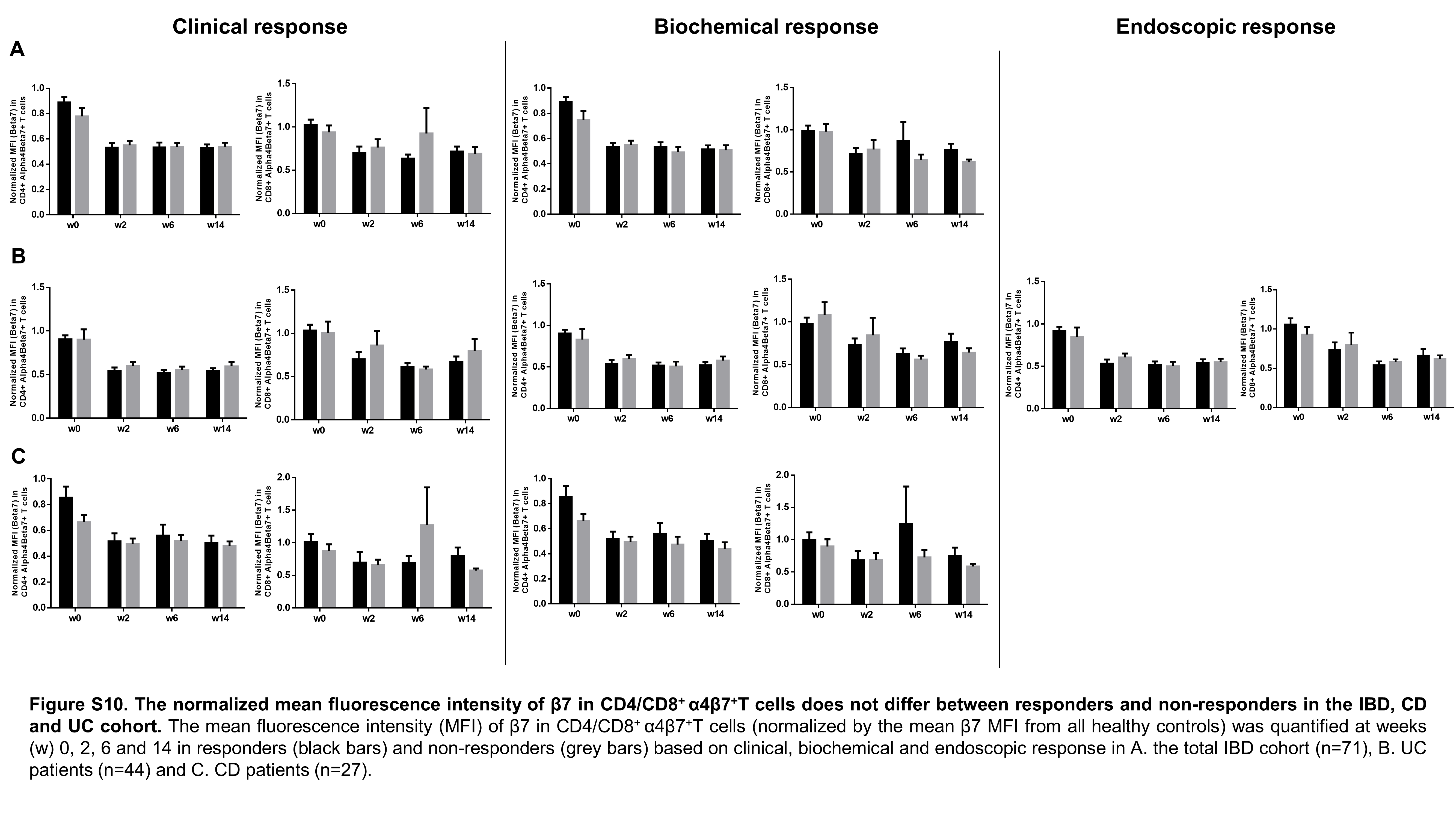

Supplement: Supplementary file 10 — SUPPORTING INFORMATION [file CTM2-12-e769-s013.tif]

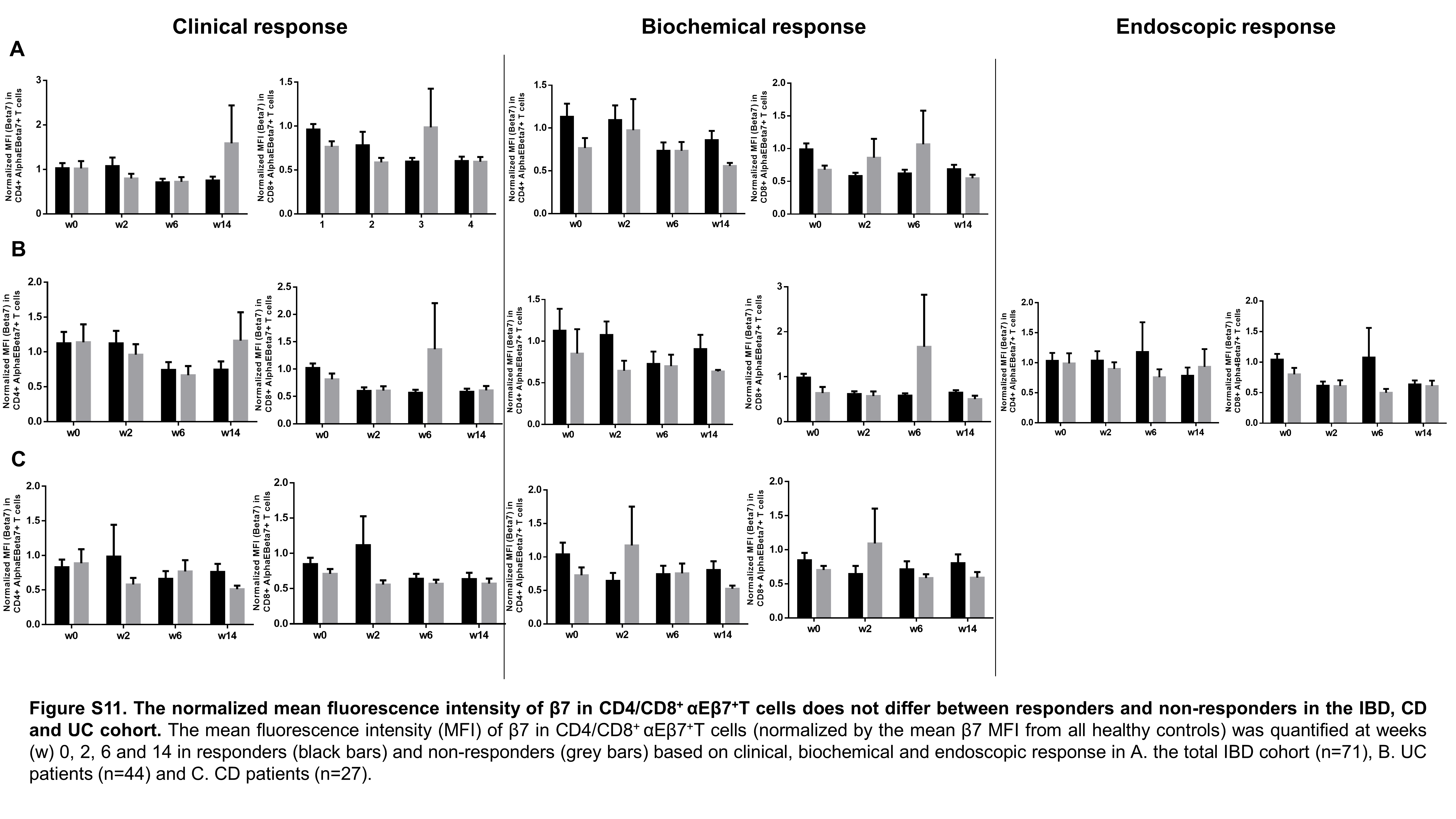

Supplement: Supplementary file 11 — SUPPORTING INFORMATION [file CTM2-12-e769-s010.tif]

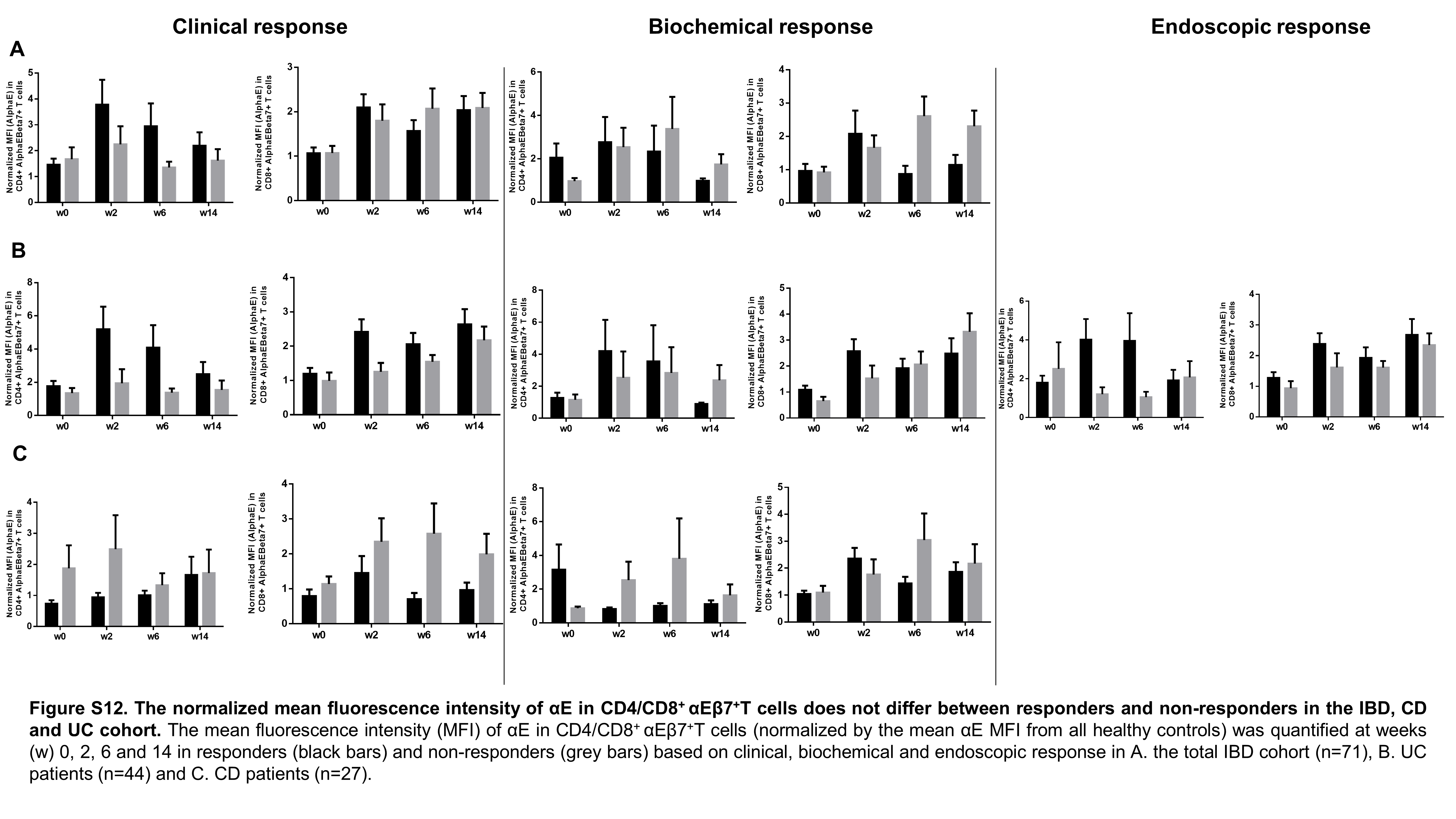

Supplement: Supplementary file 12 — SUPPORTING INFORMATION [file CTM2-12-e769-s008.tif]

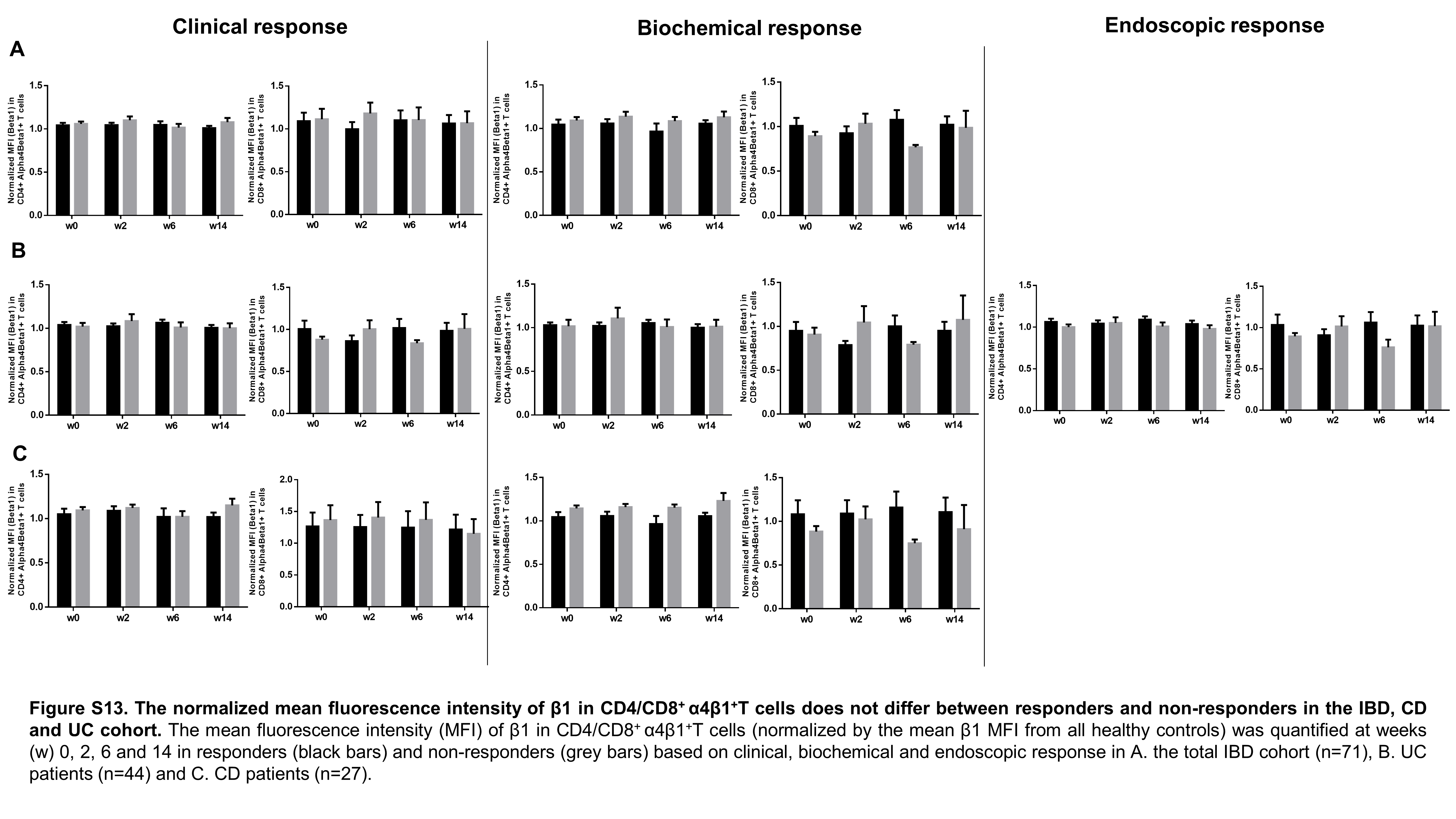

Supplement: Supplementary file 13 — SUPPORTING INFORMATION [file CTM2-12-e769-s022.tif]

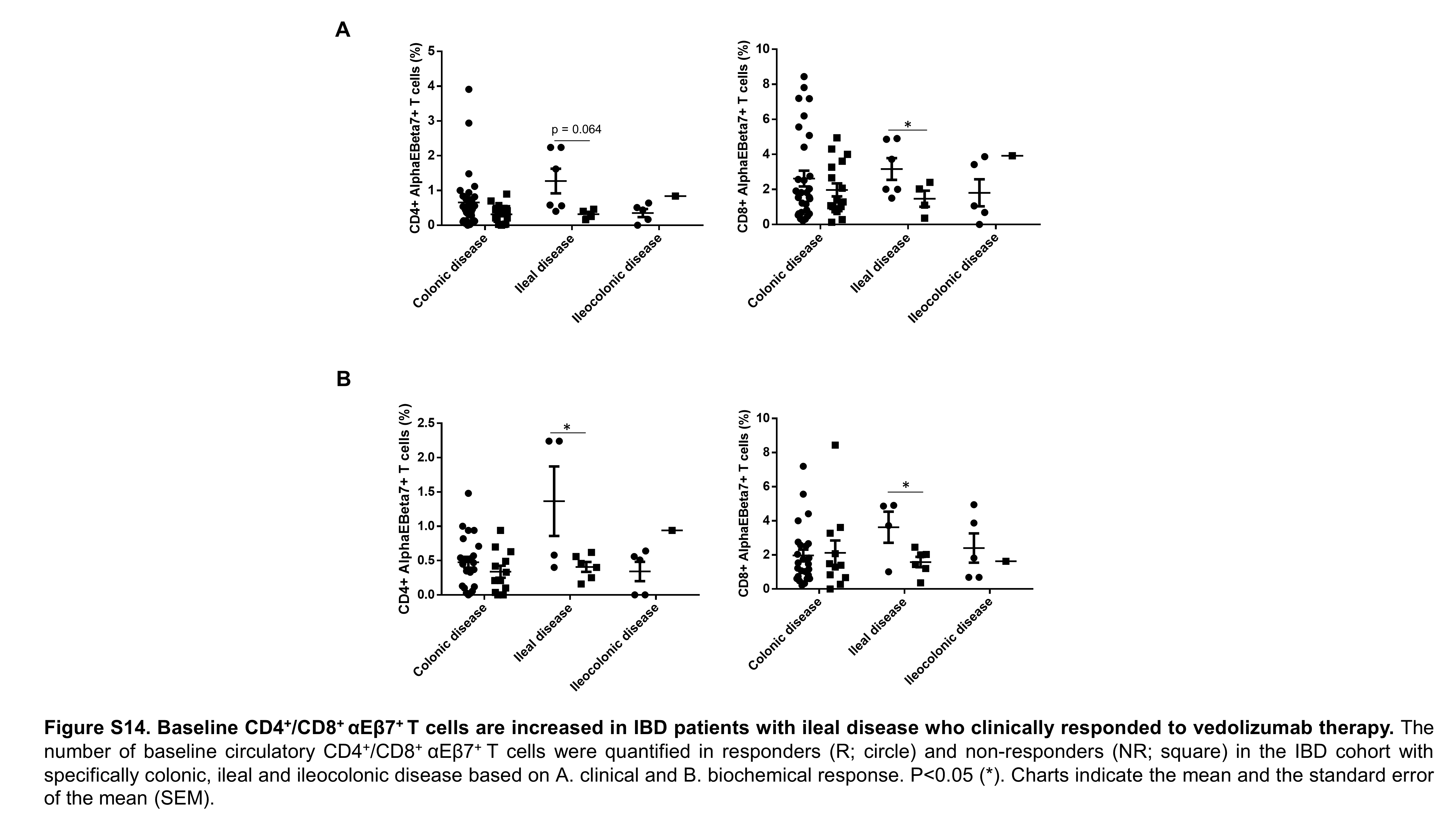

Supplement: Supplementary file 14 — SUPPORTING INFORMATION [file CTM2-12-e769-s019.tif]

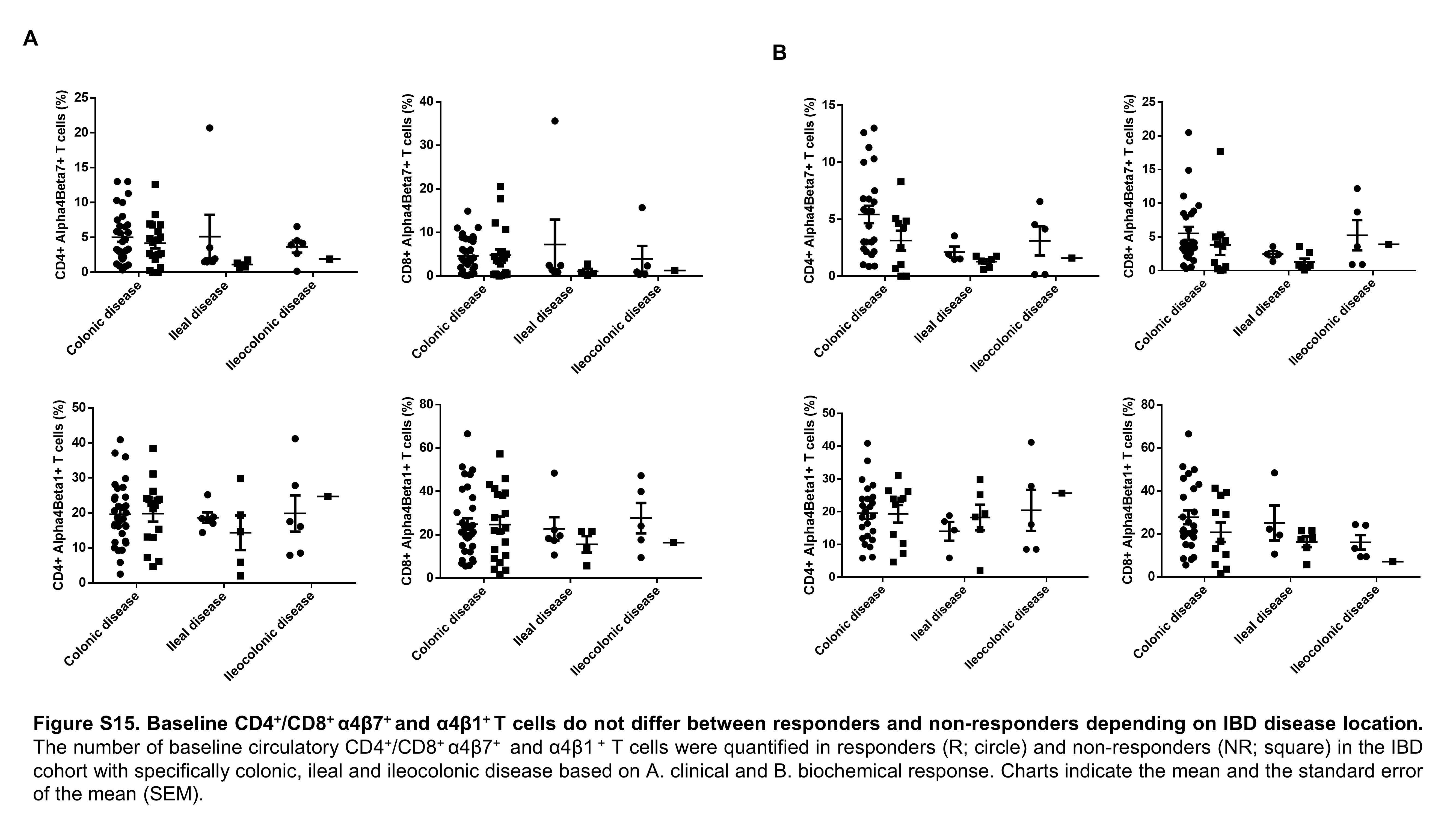

Supplement: Supplementary file 15 — SUPPORTING INFORMATION [file CTM2-12-e769-s004.tif]

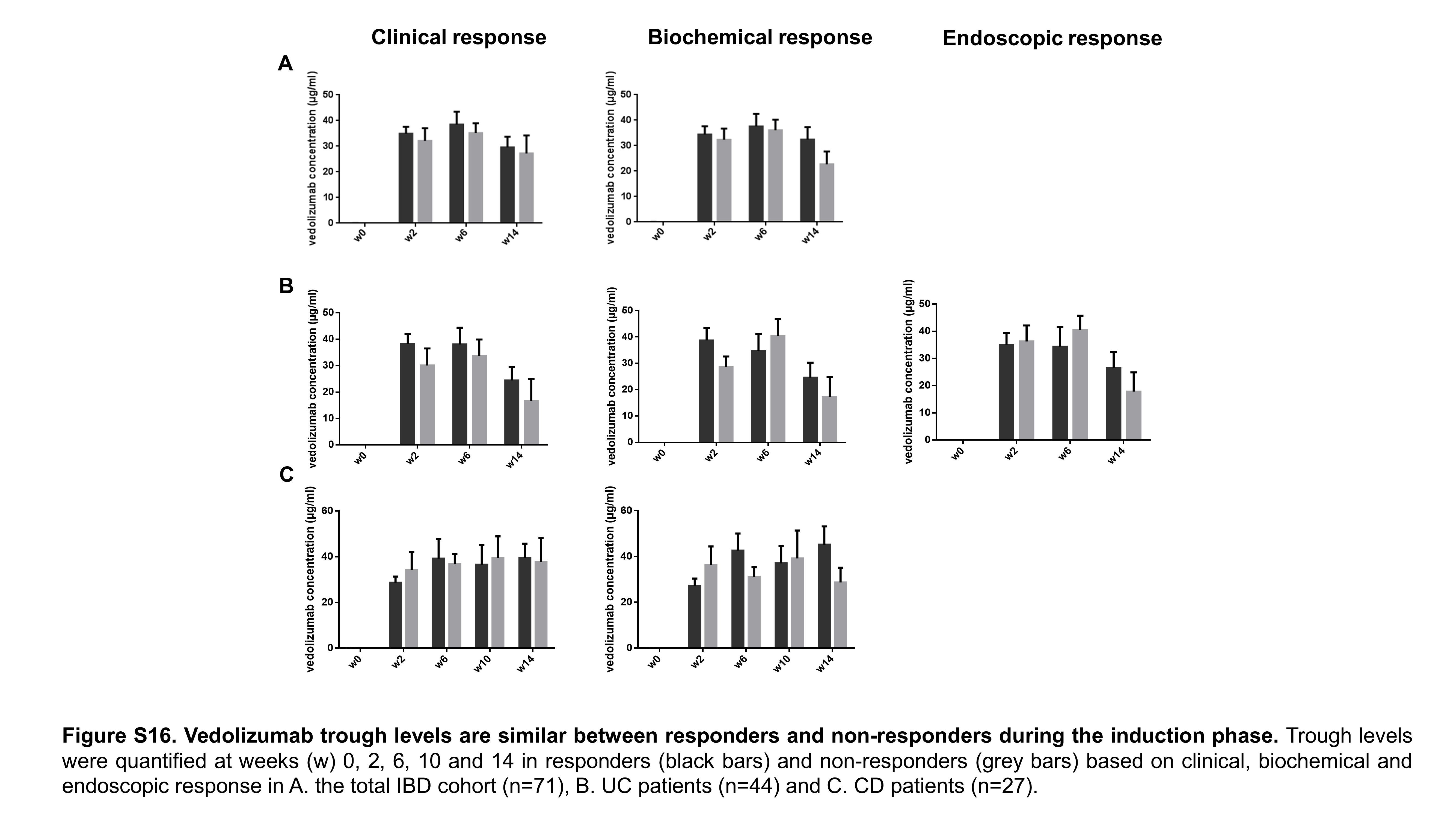

Supplement: Supplementary file 16 — SUPPORTING INFORMATION [file CTM2-12-e769-s021.tif]

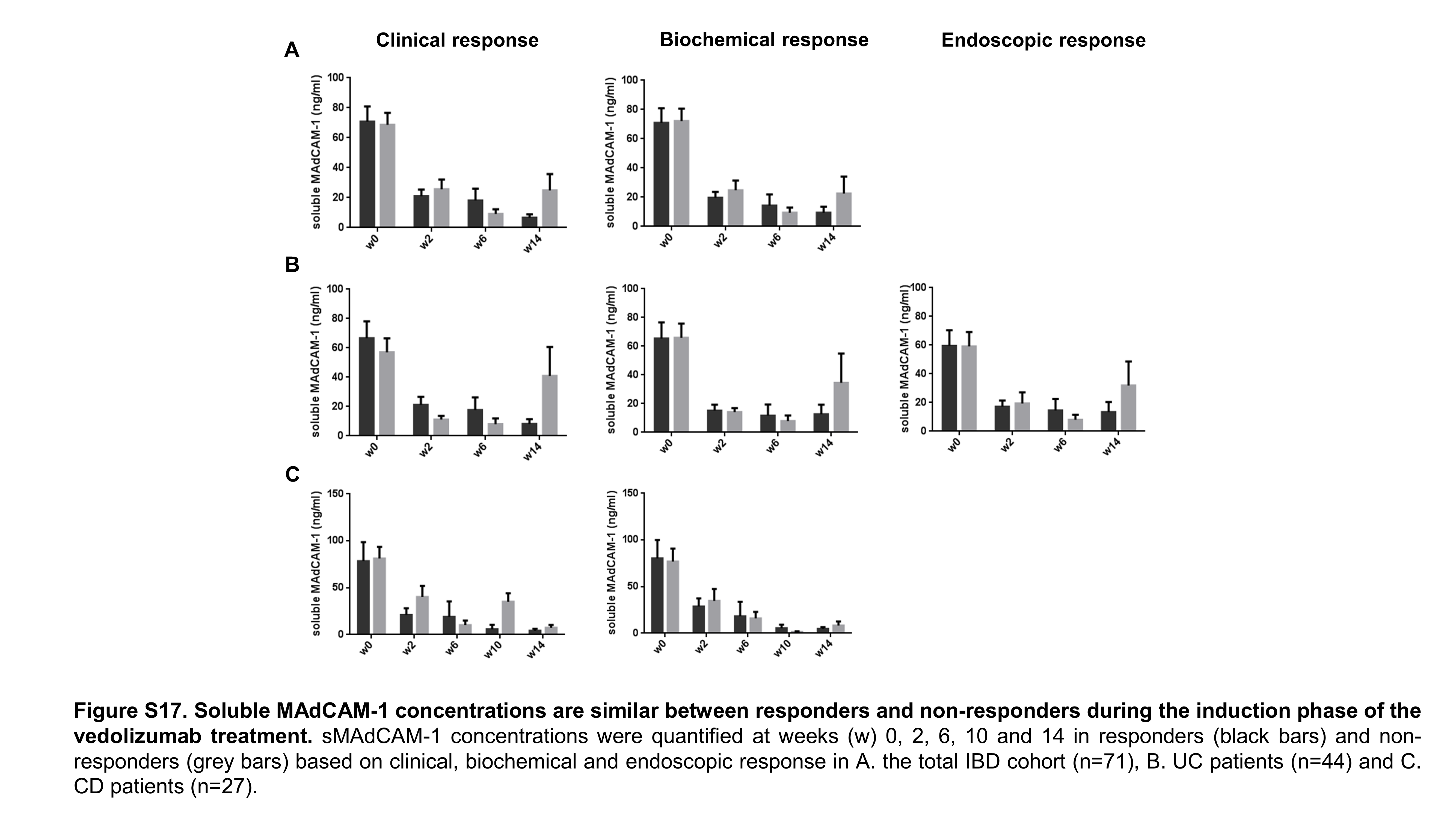

Supplement: Supplementary file 17 — SUPPORTING INFORMATION [file CTM2-12-e769-s011.tif]

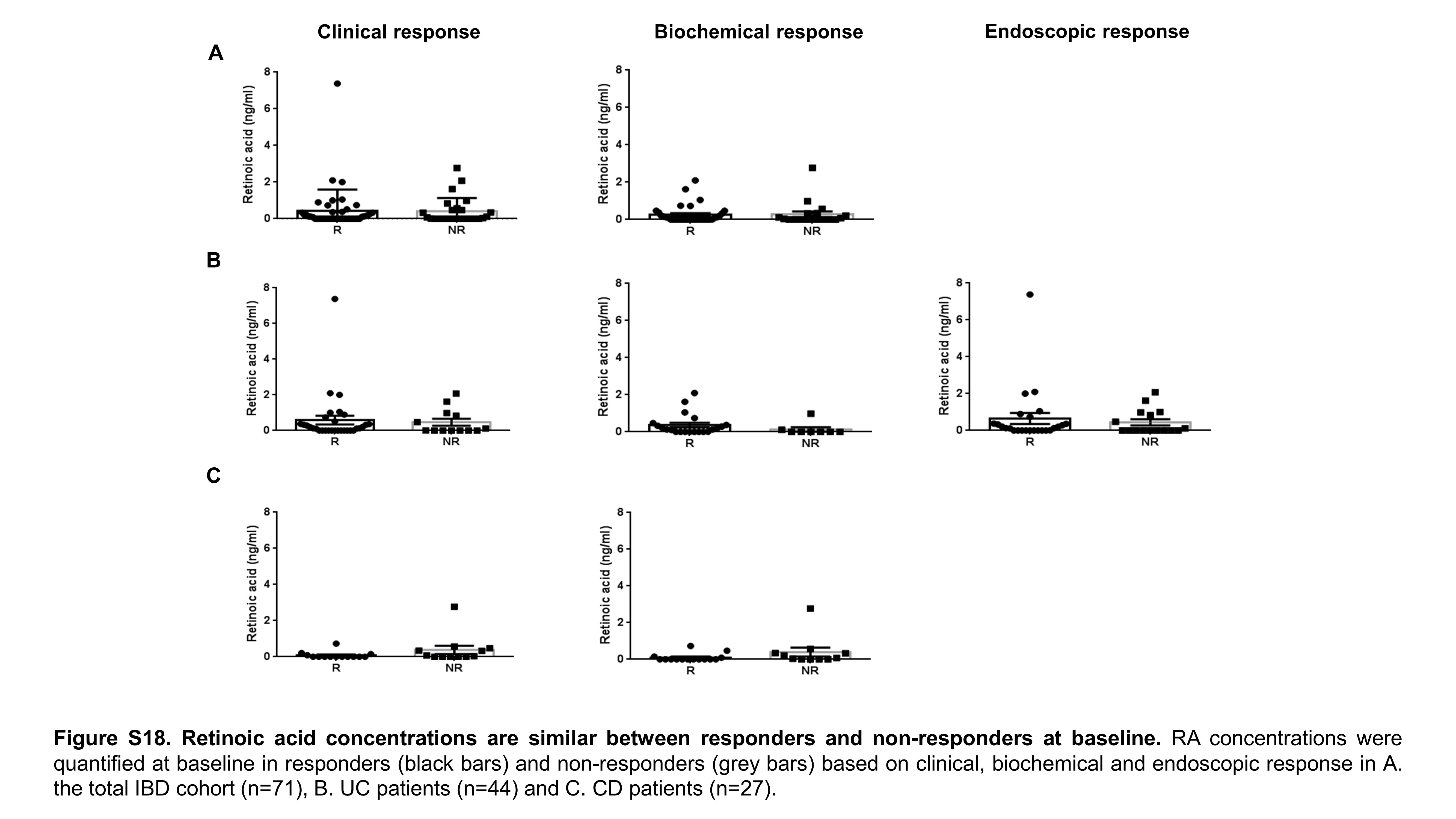

Supplement: Supplementary file 18 — SUPPORTING INFORMATION [file CTM2-12-e769-s003.tif]

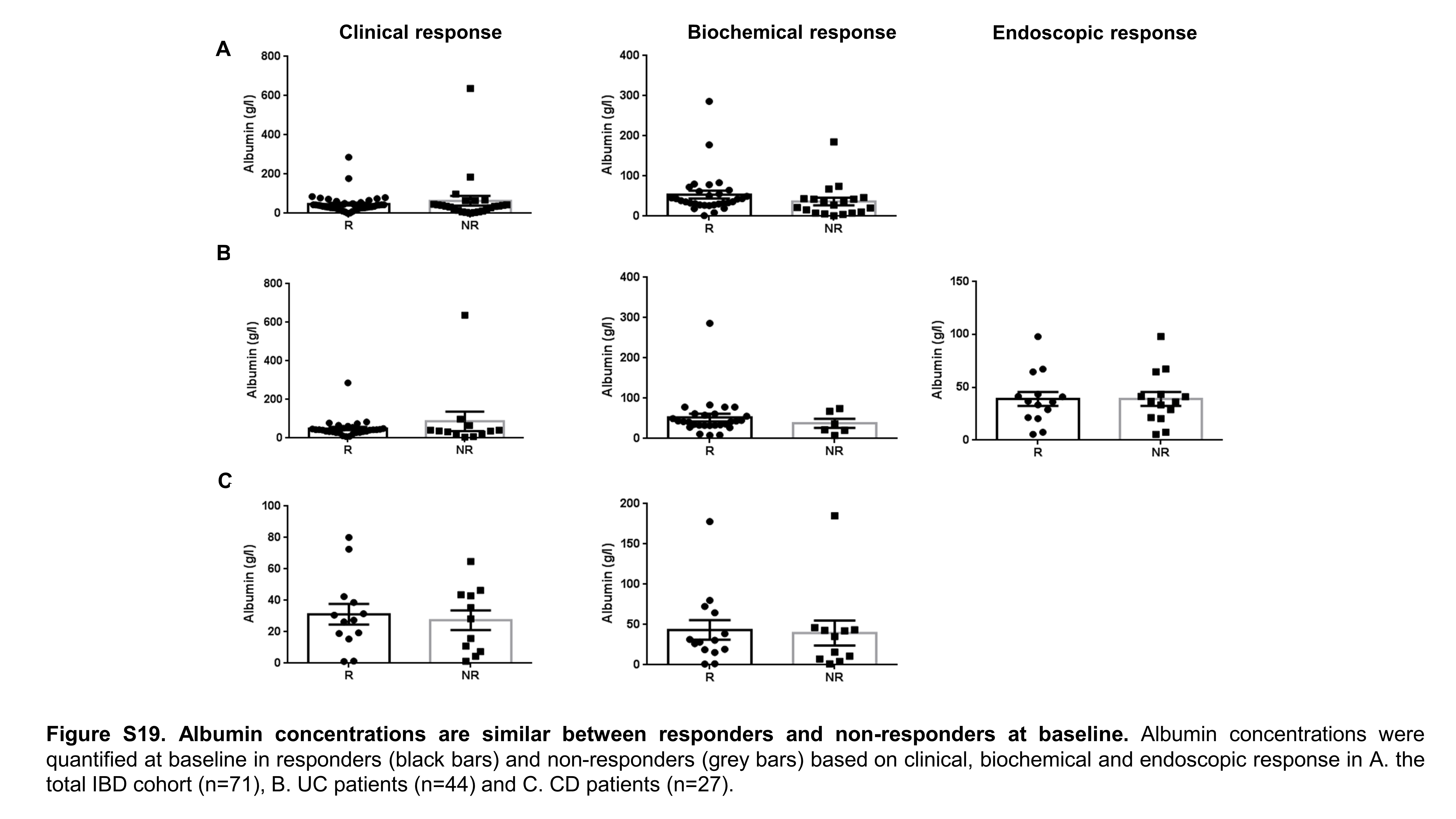

Supplement: Supplementary file 19 — SUPPORTING INFORMATION [file CTM2-12-e769-s018.tif]
