# Supplementary material for: Role of integrin expression in the prediction of response to vedolizumab: A prospective real‐life multicentre cohort study
Source: Clin Transl Med. 2022 Apr 5;12(4):e769. doi: 10.1002/ctm2.769 (PMC8982506; doi:10.1002/ctm2.769)
Supplement: Supplementary file 20 — SUPPORTING INFORMATION [file CTM2-12-e769-s016.pdf]

Table S1: Determination of vedolizumab response on a clinical, biochemical and endoscopic level in UC and CD patients at week 14

Disease \ Response	Clinical response	Biochemical response	Endoscopic response
**CD** (n=27)	HBI reduced with 3 points or more or a HBI lower than 4	50% reduction of CRP or CRP level lower than 10 mg/l	Not determined, because not part of the standard of care
**UC** (n=44)	Clinical Mayo score reduced with 3 points or more and a rectal bleeding subscore equal to 0	50% reduction of fecal calprotectin or calprotectin level lower than 250 µg/g	Endoscopic Mayo score reduced with 1 point or more

CD: Crohn's disease; CRP: C-reactive protein; HBI: Harvey Bradshaw Index; UC: Ulcerative Colitis
