# Supplementary material for: Role of integrin expression in the prediction of response to vedolizumab: A prospective real‐life multicentre cohort study
Source: Clin Transl Med. 2022 Apr 5;12(4):e769. doi: 10.1002/ctm2.769 (PMC8982506; doi:10.1002/ctm2.769)
Supplement: Supplementary file 21 — SUPPORTING INFORMATION [file CTM2-12-e769-s005.pdf]

Table S2: Characteristics of the study population

	Total n=71†	CD n=27	UC n=44	*p*
**Age** (years), mean [min - max]	41 [18 – 76]	41 [20 – 68]	42 [18 – 76]	ns
**Gender**, n [%]				ns
Female	40 [56.3]	15 [55.6]	25 [56.8]	
Male	31 [43.7]	12 [44.4]	19 [43.2]	
**Age at diagnosis**, mean [min - max]	32 [11 – 68]	32 [11 – 64]	33 [11 – 68]	ns
**Length** (m), median [IQR]	1.7 [1.6 –1.8]	1.7 [1.6 – 1.8]	1.7 [1.6 – 1.8]	ns
**Weight** (kg), median [IQR]	67 [60 – 80]	69 [59 – 80]	66 [60 – 81]	ns
**Smoking**‡, n [%]				ns
Yes	18 [25.4]	9 [33.3]	9 [20.5]	
No	53 [74.6]	18 [66.7]	35 [79.5]	
**Disease localization**, n [%]				*
Proctitis	2 [2.8]	0 [0.0]	2 [4.5]	
Left-sided colitis	28 [38.0]	2 [7.5]	26 [59.1]	
Extensive colitis	20 [28.2]	8 [29.6]	16 [36.4]	
Ileocolonic	14 [19.7]	5 [18.5]	0 [0.0]	
Ileal	7 [11.3]	12 [44.4]	0 [0.0]	
**Previous surgery**, n [%]	7 [9.9]	7 [25.9]	0 [0.0]	ns
**Previous anti-TNF use** [%]	38 [53.5]	12 [44.4]	26 [59.1]	ns
**Concomitant drug at week 0** [%]				
5-ASA	32 [45.1]	2 [7.4]	30 [68.2]	***
Steroids	23 [32.4]	9 [33.3]	14 [31.8]	ns
Azathioprine	13 [18.3]	8 [29.6]	5 [11.4]	ns
Methotrexate	1 [1.4]	1 [3.7]	0 [0.0]	ns
**Concomitant drug at week 14** [%]				
5-ASA	32 [45.1]	1 [3.7]	31 [70.5]	***
Steroids	2 [2.8]	1 [3.7]	1 [2.3]	ns
Azathioprine	15 [21.1]	7 [25.9]	8 [18.2]	ns
Methotrexate	1 [1.4]	0 [0.0]	1 [2.3]	ns

†N_{UZG}=48; N_{AZSL}=6; N_{AZG}=16; N_{UZA}=1; ‡Current and previous history of smoking; 5-ASA: 5-aminosalicylic acid;

*p<0.05; ***p<0.001; ns: not significant
