# Supplementary material for: Role of integrin expression in the prediction of response to vedolizumab: A prospective real‐life multicentre cohort study
Source: Clin Transl Med. 2022 Apr 5;12(4):e769. doi: 10.1002/ctm2.769 (PMC8982506; doi:10.1002/ctm2.769)
Supplement: Supplementary file 22 — SUPPORTING INFORMATION [file CTM2-12-e769-s020.pdf]

Table S3: Patient reported outcomes

	Clinical response		*p*	Biochemical response		*p*	Endoscopic response		*p*
	**R**	**NR**		**R**	**NR**		**R**	**NR**	
**UC**	30 [68.18]	14 [31.82]	ns	21 [72.41]	8 [27.59]	ns	25 [60.98]	16 [39.02]	NA
**CD**	14 [51.85]	13 [48.15]		15 [55.56]	12 [44.44]		NA	NA	
**Total**	44 [61.97]	27 [38.03]		36 [64.29]	20 [35.71]		25 [60.98]	16 [39.02]	

R: responders, NR: non-responders, [%], NA: not applicable, ns: not significant.
