# Supplementary material for: Role of integrin expression in the prediction of response to vedolizumab: A prospective real‐life multicentre cohort study
Source: Clin Transl Med. 2022 Apr 5;12(4):e769. doi: 10.1002/ctm2.769 (PMC8982506; doi:10.1002/ctm2.769)
Supplement: Supplementary file 23 — SUPPORTING INFORMATION [file CTM2-12-e769-s009.pdf]

SUPPLEMENTARY METHODS

Patient inclusion and study design

The PROVE-IBD study is a prospective multicentre observational study. The study cohort (n=71) consisted of 44 patients with moderate-to-severe UC and 27 patients with moderate-to-severe CD, who initiated vedolizumab as part of their conventional treatment plan and fulfilling the national reimbursement criteria (which entails previous failure of immunosuppressants). Active CD was defined by a Harvey-Bradshaw Index (HBI) score higher than 4 together with a C-reactive protein (CRP) level higher than 10 mg/l or fecal calprotectin higher than 250 µg/g or presence of ulcers during endoscopy. Active UC was defined as a total Mayo score higher than 7. All subjects were at least 18 years of age at the initiation of vedolizumab therapy. Exclusion criteria were unclassified IBD, previous treatment with anti-adhesion molecules and subtotal colectomy or proctocolectomy prior to vedolizumab initiation. The concomitant use of thiopurines or steroids was allowed in case of a stable dose for at least 4 and 2 weeks, respectively, however the use of steroids was tapered at week 10 to assure that the response defined at week 14 could be attributed to vedolizumab treatment. According to the treatment guidelines, patients received 300 mg intravenous vedolizumab at weeks 0, 2 and 6, while CD patients received an additional dose at week 10. Some patients did not follow the full treatment plan due to the development of complications during the induction phase. Data obtained from these patients were included using the *last observation carried forward* method.

Data and sample collection

All patients were screened at baseline (week 0) by determining the Clinical Mayo score and the endoscopic Mayo subscore for UC and the HBI for CD. Patient demographic

and clinical characteristics were collected based on questionnaires and included age, gender, age at diagnosis, height, weight, disease location, the history of medical/surgical treatment of IBD, previous use of TNF antagonists and smoking habits (Table S2). All participants received a stool collection vial for at-home sampling (max. 24h before vedolizumab administration). Patients were instructed to provide 3 stool samples, one collected before the initiation of vedolizumab treatment (week 0), a second collection before the third infusion (week 6) and at week 14. All samples were kept at 4°C until delivery to the laboratory and stored at -80°C. Serum and EDTA blood was collected before each vedolizumab infusion, to avoid integrin heterodimer α4β7 internalization by the drug, which would interfere with flow cytometry staining. Serum was stored at -20°C, and peripheral blood mononuclear cells (PBMCs) were isolated from EDTA blood (6h after collection to reduce variation that could be attributed to the timing between sampling and cell isolation (Figure S1)).

Quantification of biochemical markers

Fecal calprotectin was measured within 4 days of storage using the Quantum Blue[R] fCAL kit (Buhlmann Laboratories AG, Shonenbuch, Switzerland) following the manufacturer's instructions. Serum CRP was measured using the Luminex[R] CRP kit (R&D Systems, Minnesota, USA). Trough levels were quantified using the vedolizumab trough level assay (Apdia, Turnhout, Belgium)[1,2], serum sMAdCAM-1 concentrations were measured using the sMAdCAM-1 ELISA kit (Hycult Biotech, Uden, Netherlands)[3], serum RA concentrations were quantified using the Human Retinoic Acid ELISA kit (MyBioresource, California, USA) and baseline serum albumin concentrations were quantified using the Albumin Human ELISA kit (Thermo Fisher, Massachusetts, USA).

PBMC isolation and flow cytometry

Twenty ml of whole blood was used to isolate PBMCs using Leucosep tubes (Greiner Bio-One, Brussels, Belgium). Cells were stored in cold RPMI medium containing 0.3 g/l glutamine, 1% penicillin, 10% fetal calf serum (FCS) (Thermo Fisher, Massachusetts, USA) and 10% dimethylsulfoxide (Sigma-Aldrich, Missouri, USA) at -180°C at the Bioresource center Ghent (Ghent, Belgium, ID: BE 71067049). For flow cytometry, PBMCs were thawed in a warm water bath (37°C), washed and resuspended in 10 ml warm RPMI containing 10% FCS. The PBMCs were then incubated for 30 minutes at 37°C and washed a second time. Next, PBMCs were incubated with anti-human BD Fc block (BD, New Jersey, USA) and labelled with Alexa fluor 700 anti-human CD3 (clone: SK7, Biolegend, California, USA), Pacific Blue anti-human CD4 (clone: SK3, Biolegend, California, USA), FITC mouse anti-human CD8 (clone: HIT8α, BD, New Jersey, USA), PerCP-Cy5.5. mouse anti-human CD49d (clone: 9F10, BD, New Jersey, USA), BV605 rat anti-integrin Beta7 (clone: FIB504, BD, New Jersey, USA), PE mouse anti-human CD29 (clone: MAR4, BD, New Jersey, USA), PE-Cy7 anti-human CD103 (clone: Ber-ACT8, Biolegend, California, USA) and eBioscience Fixable viability dye efluor 506 (Thermo Fisher, Massachusetts, USA). Batch variations were reduced using identical application settings with a fixed compensation matrix. To correct for batch effects, we included PBMCs from 3 healthy controls and Vericells (Biolegend, California, USA) in each run. Rainbow calibration particles (Spherotech, Illinois, USA) were used to monitor technical variation over time. Gating was performed using fluorescence minus ones (FMO's) for CD49d, integrin Beta7, CD29 and CD103 on each plate (for gating strategy, Figure S2 and S3). Flow cytometry analysis was performed on the FACS Fortessa (BD, New Jersey, USA) and data were analysed in Flowjo version 9. Before analysing the patient samples, we

confirmed that the presence of vedolizumab did not inhibit the binding of the antibodies targeting integrins α4 and β7 (Figure S4).

Statistical analysis

Univariate analyses were performed in SPSS statistics version 25. Baseline differences in flow cytometry profile, sMAdCAM-1, trough levels, RA and albumin concentrations were determined using independent sample t-test (or Mann-Whitney U test in case of violations of the normality distribution assumption). The difference in delta changes were assessed using one-way ANOVA. False discovery correction was performed using the Benjamini and Hochberg multiple comparison correction. Next, demographic differences were identified by a Fisher's exact test.

A predictive model was developed using both elastic net regularized regression (EN) and random forest (RF) including flow cytometry, trough level, sMAdCAM-1, RA concentration, biochemical markers and demographic data as independent variables, and clinical response as dependent variable. The EN model was implemented using the "glmnet" package in R. EN is an integration of two regularized approaches, least absolute shrinkage and selection operator (LASSO) and ridge regression, where the contribution of each of these models to the final EN model is controlled by the α parameter. The ridge regression component of EN addresses potential multi-collinearity problems in the data whereas the penalization imposed by LASSO draws coefficients to zero thereby eliminating non-predictive variables. The penalization parameter λ was determined by estimating the area under the receiver operator curve (AUROC) of the population using five-fold cross-validation. Several α parameter values were assessed (0.25, 0.5, 0.75, 1.0). Validation of the model was performed via bootstrapping using the BootValidation package in R with 2000 iterations. Predictors

were considered robust and reliable when they appear in at least 80% of all model iterations. The data was also split into 70:30 ratio for training and validation set, respectively, to further validate the predictive ability of the model. The predictive model was built using the training set and the validity of the model was assessed using the validation set. Model validation was based on AUROC and a cut-off of 70% was considered for validation. On the other hand, RF was performed using “ranger” package in R with 500 trees. Similarly, the model was built on the training set and a cut-off of 70% AUROC on the validation set was used.

Data availability statement

The data that support the findings of this study are available from the corresponding author upon reasonable request.

Ethical considerations

This study was approved by the Ethical committee of the University Hospital of Ghent (EC2016/1366) and was performed in accordance with the Declaration of Helsinki (1964, and its later amendments). Written informed consent was obtained from all participants prior to their inclusion in the study.

References for supplementary methods

1. Verstockt B, Verstockt S, Veny M, Dehairs J, Arnauts K, Van Assche G, De Hertogh G, Vermeire S, Salas A, Ferrante M. Expression levels of 4 genes in colon tissue might be used to predict which patients will enter endoscopic remission after vedolizumab therapy for inflammatory bowel diseases. *Clin Gastroenterol Hepatol* 2019;12:1142-1151*.*
2. Van den Berghe N, Verstockt B, Tops S, Ferrante M, Vermeire S, Gils A. Immunogenicity is not the driving force of treatment failure in vedolizumab-treated inflammatory bowel disease patients. *J Gastroenterol Hepatol* 2018;34:1175-1181.
3. Paul S, Williet N, Di Bernado T, Berger AE, Boschetti G, Fillipi J, Del Tedesco E, Nancey S, Flourie B, Roblin X. Soluble mucosal addressin cell adhesion molecule 1 and retinoic acid are potential tools for therapeutic drug monitoring in patients with inflammatory bowel disease treated with vedolizumab: a proof of concept study. *J Crohns Colitis* 2018;12:1089-1096.
